# The State Board of Health Matter

**Published:** 1903-02

**Authors:** 


### THE TEXAS MEDICAL JOURNAL.

AUSTIN, TEXAS.

A MONTHLY JOURNAL OF MEDICINE AND SURGERY.

EDITED AND PUBLISHED BY
F. E. DANIEL, M. D.

ASSOCIATE EDITORS:

WITTEN BOOTH RUSS, M. D., San Antonio, Texas.

P. M. PAYNE, M. D., Brownwood, Texas.

Published Monthly at Austin, Texas. Subscription price $1.00 a year in advance.

Eastern Representative: John Guy Monihan, St. Paul Building, 220 Broadway, New York City.

Official organ of the the West Texas Medical Association, the Houston District Medical Association, the Austin District Medical Society, the Brazos Valley Medical Association, the Galveston County Medical Society, and several others.

### THE STATE BOARD OF HEALTH MATTER.

In the January number of the "Red Back," I published a bill that had been prepared by the chairman of the State Medical Association's committee and submitted to the committee. They could not agree upon it, and the chairman tendered his resignation. It was not accepted, and was withdrawn. Meantime no member of the committee moved in the matter—no other bill was prepared, and it seemed that the committee were going to let it go by default. The Legislature had met meantime, and it was necessary to act. The chairman a second time requested a meeting of the committee. It was called for January 21—the day after the inauguration of the Governor—in the hope of getting a full attendance. No member, outside of Austin, put in an appearance except Dr. Coleman, of Mitchell county, whose home is several hundred miles away. Dr. Daniel (Chairman), Dr. Tabor, State Health Officer, and Dr. Coleman had a conference, and Dr. Douglas, Senator from Hill county, and chairman of the Senate Public Health Committee, was present by invitation. The result of the conference and a conference with a number of members of both houses was the subjoined bill. We are satisfied that it is the very best that can be done, and we have high hopes that it will pass without material change. This bill was introduced in the Senate by Senator Harper, of Brazos county, on 4th inst., and at this writing is before the Senate Committee on Public Health. Drs. Daniel and Tabor will be given a hearing before the committee this week, and perhaps before this goes to press.

It will be seen, upon a careful examination of the bill, that it accomplishes all that it was hoped to accomplish by a board of health, and by a less cumbersome and expensive organization. It perfects the machinery and provides for a very comprehensive system of sanitation.

The bill is commended to the medical profession of Texas, and the signing members of the committee ardently hope to have their approval and co-operation in the effort to have it enacted and put in operation.

To this end the undersigned members of the committee call upon and urge every member of the State Association and all other physicians who appreciate the importance and necessity of such a law to *at once,* without delay, write to their immediate representatives in both houses, insisting upon their support of the bill.

The bill is entitled:

AN ACT

To carry into effect Section 32, of Article 16, of the Constitution of the State of Texas in relation to vital statistics; to enlarge the scope and powers of the existing health system of the State for the better protection of the public health; to change the name of the Quarantine Department to the Department of Public Health and Vital Statistics, and to create and establish a State Bureau of Vital Statistics within said department; to authorize the State Health Officer to prepare, promulgate and enforce, under suitable penalties for the violation of its provisions, a sanitary code for the State of Texas, and regulations for the record and preservation of its vital statistics, and to repeal all laws and parts of laws in conflict with this act.

SECTION 1. *Be it enacted by the Legislature of the State of Texas:* That a Bureau of Vital Statistics is hereby created and established within the Quarantine Department, and that the name of said department is hereby changed to the Department of Public Health and Vital Statistics.

SEC. 2. The State Health Officer shall, by and with the approval of the Governor, after the passage of this act, appoint from among the duly qualified physicians of the State, one who is learned in sanitation and familiar with statistics, who shall be registrar of vital statistics, who shall, under the direction and supervision of the head of the department, collect, record, tabulate and publish in the yearly report of the department the vital and mortuary statistics of the State, and who shall be paid a salary of two thousand dollars per annum.

SEC. 3. All physicians, surgeons or accoucheurs who may attend at the birth of a child; or in the absence of such attendance, either parent of the child, or any other party having knowledge of the same, shall report the fact to the clerk of the county court, together with the race to which the child belongs, and whether legitimate or otherwise, or foreign or native parents, whether still-born or alive, within ten days after said birth occurs, under a penalty of five dollars for each failure to do so; to be collected as other fines for misdemeanors are.

All physicians, surgeons, accoucheurs, coroners, or in the absence of these, any person who shall become cognizant of a death, shall report the same, together with the race, nativity, sex, age, residence, whether alien or citizen, and the cause of death, to the clerk of the county court within ten days after the occurrence, under a penalty of not less than five dollars nor more than fifty dollars for each failure to do so; these data to be recorded as a part of the vital statistics of the county and State, and the clerk of the court shall report monthly all these data to the Department of Public Health and Vital Statistics. In default of so reporting he shall be fined not less than fifty dollars for each offense.

SEC. 4. The State Health Officer shall prepare a sanitary code for the State of Texas, which shall contain and provide rules and regulations, and ordinances of a general nature, for the improvement and amelioration of the hygienic and sanitary conditions of the State.

There shall be printed and published in pamphlet form such number of copies as may be necessary, for distribution for the information of health boards, health and sanitary officers, and the public generally. Said code shall cover and provide for, especially, land and maritime quarantine regulations; the reporting, care and management of infectious, contagious and communicable diseases; it shall regulate the manner of keeping, reporting and tabulating vital and mortuary statistics; it shall provide for affording facilities for vaccination, provided the same shall not be made compulsory except in cases of children attending public schools; it shall regulate the carriage and transportation of persons, freight and dead bodies in, or brought into the State, or transported through or into the State, so far as the same may affect the public health; it shall provide for the carrying out of the laws of the State in regard to the adulteration of articles intended for human food or consumption; it shall provide for the inspection of meats, milk, coal oil and other articles affecting the public health and safety where and when the same may be brought from one county into another, or from outside the State, leaving to local boards the regulation of the sale, or offering for sale, of said articles within the county or municipality to which the same may be brought; and said code shall contain general rules in regard to such health, sanitary and hygienic subjects as can not in the opinion of the State Health Officer be efficiently and effectively regulated by the local boards; it shall devise under suitable penalties for violation thereof ordinances to prevent the pollution of all water supplies and streams; it shall contain, and the head of the department shall have power to enforce, under suitable penalties, ordinances to regulate sanitary policing of premises, public or private, jails, hospitals, public buildings, shops, factories, slaughter houses and yards, for the removal of possible causes of disease; to abate nuisances, to cause disinfection of all infected houses, public or private, and of sleeping cars and other railroad cars, and of all public conveyances, this sanitary policing and means of prevention to be done by the owners, or agents, of said premises or conveyances at their own cost.

SEC. 5. The State Health Officer shall have power to appoint inspectors when necessary, and fix the salaries thereof. The inspectors and officers shall have power to arrest, without warrant, all persons violating the provisions or any rule or regulation thus prescribed by the public health department, when such violation has occurred, or is occurring, within the sight, view or personal knowledge of such inspector or officer, and in all cases where such violation may not have occurred within the sight, view or personal knowledge of such inspector or officer, said functionary shall have only the right to arrest in execution of a warrant duly issued by the State Health Officer.

It is hereby made the duty of all sheriffs, and their deputies, police officers of towns and cities, and all other peace officers, to assist in the arrest or apprehension of all persons violating the articles or any rule or regulation of the Public Health Department, and to themselves arrest and apprehend all offenders committing offenses in their view or sight, or within their personal knowledge.

SEC. 6. In the event that any case shall be reported to or come to the knowledge of any local board or health officer, which is either deemed to be a case of contagious or infectious disease, or is suspected of so being, said board, or health officer, shall immediately isolate the same and communicate the fact to the Public Health Department,. together with the information as to what has been done toward the isolation and care of the same, and shall from time to time communicate the progress of the case and disease to the Public Health Department. On receipt of such information the State Health Officer shall. if he deem the emergency sufficient and necessary, visit in person or send the Assistant State Health Officer, hereinafter provided for, or some other regular physician, expert in diagnosis, to examine and diagnose the disease; and if on such examination and diagnosis he shall declare the case to be one of contagious or infectious nature, liable to spread or become dangerous to the public health of the State, the State Health Officer shall instruct the health officer, or the board of health of the county, or town, as to what steps to take, if any shall be taken, for the further management of the case to prevent the spread of the contagion or infection therefrom, and shall require that the local health officer shall immediately conform to and put the same in operation. In the event that said local authorities shall fail or neglect to so act promptly, or in so acting shall fail to do so in a manner satisfactory to the Public Health Department, the said health officer shall take charge of the case and manage the same through the officers or employes of the department.

SEC. 7. The State Health Officer shall appoint from among the qualified physicians of the State one who is learned in sanitation, and expert in diagnosis, to be Assistant State Health Officer; his duties shall be that of a general assistant to the State Health Officer, and he shall receive an annual salary of eighteen hundred dollars. The State Health Officer shall employ a clerk, who shall be bookkeeper and stenographer for the department; he shall receive a salary of twelve hundred dollars per annum.

County judges and mayors of cities are hereby required to furnish to the State Health Officer the names of their county and city physicians as soon as they are appointed, as is required in the existing quarantine law. In default of so reporting within thirty days after appointment, said county judges, or mayors, may be fined not less than five dollars, nor more than fifty dollars for each offense.

SEC. 8. The salaries of the officers and employes, and all other expenses provided for in the foregoing, including two thousand dollars or as much thereof as shall be necessary for the services of a chemist, as authorized by the provisions of Article 4317, Title

XCI, page 865, Revised Statutes of 1895, shall be paid out of the appropriation for the Department of Public Health and Vital Statistics.

SEC. 9. All laws and parts of laws in conflict with, inconsistent with, or superceded by the provisions of this act, are hereby repealed; but all laws or parts of laws, or city ordinances, or laws of the State, and municipal rules and regulations now existing, and not in conflict with, or inconsistent with, or superceded by the provisions of this act, are continued in full force and effect, and this act shall not be so construed or interpreted as to deprive the State Health Officer or local boards of health of any powers or authority they may have under existing laws, except in so far as these powers and authorities may be modified or changed by the provisions of this act, and then only to the extent of the modification or change.

The fact that there is no uniform and efficient law for the suppression of diseases, other than those of foreign origin, and no provision for or system of preserving the vital statistics of the State, creates an emergency and an imperative public necessity that the constitutional rules requiring bills to be read on three several days be suspended, and that this act take effect and be enforced from and after its passage, and it is so enacted.

F. E. DANIEL,
BACON SAUNDERS,
P. C. COLEMAN,
GEO. R. TABOR,
Committee.

Dissenting:

F. PASCHAL,
W. R. BLAYLOCK.

